# Understanding Surface Properties in CeO_2_ Catalysts for the Synthesis of Dimethyl Carbonate: A Combined In Situ IR and NEXAFS Study

**DOI:** 10.1021/acs.jpcc.5c04979

**Published:** 2025-10-23

**Authors:** Gionata Galliano, Edoardo Bracciotti, Andrea Jouve, Luca Braglia, Rudy Calligaro, Elisa Borfecchia, Sergio Rojas-Buzo, Silvia Bordiga

**Affiliations:** a Department of Chemistry, NIS Center and INSTM Reference Center, 9314 University of Turin, Turin 10125, Italy; b Area Science Park, Padriciano 99, Trieste 34149, Italy; c CNR-IOM, Strada Statale 14 Km 163.5, Basovizza, Trieste 34149, Italy; d Dipartimento Politecnico, 9316 Università degli Studi di Udine, Via del Cotonificio 108, Udine 33100, Italy; e IIQ, Instituto de Investigaciones Químicas (CSIC-Universidad de Sevilla), Avda. Americo Vespucio 49, Seville 41092, Spain

## Abstract

In this work, we studied the surface properties of two different CeO_2_ catalysts, one synthesized by a modified hydrothermal method and the other obtained by a nonconventional calcination of a metal–organic framework (MOF). The first one presented a high surface area (CeO_2_-HSA) with accessible Ce sites located primarily on (111) planes, while the MOF-derived material (CeO_2_-MOF) showed coordinatively unsaturated Ce sites (CUS) located on (110) planes. *In situ* IR and NEXAFS spectroscopies were employed to unravel the nature of the surface intermediates and the Ce oxidation state during the reaction. Both materials show Ce reduction during the adsorption of methanol as a consequence of methoxide-to-formate decomposition, while CeO_2_-HSA produces a high proportion of surface HCOO-Ce^3+^ as a consequence of its higher surface area. However, as we reported previously, this high proportion of surface Ce^3+^ sites causes catalyst deactivation. In this sense, CeO_2_-MOF presented a high concentration of CUS sites located on (110) planes, which are beneficial for the direct synthesis of DMC from CO_2_ and methanol.

## Graphical abstract

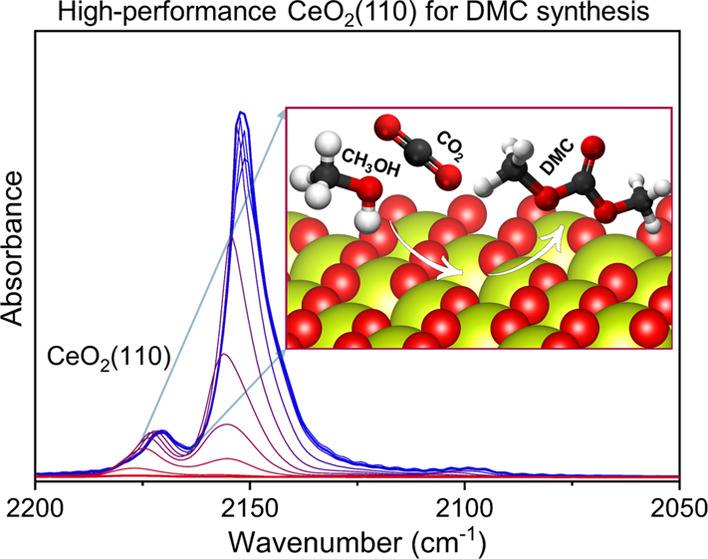

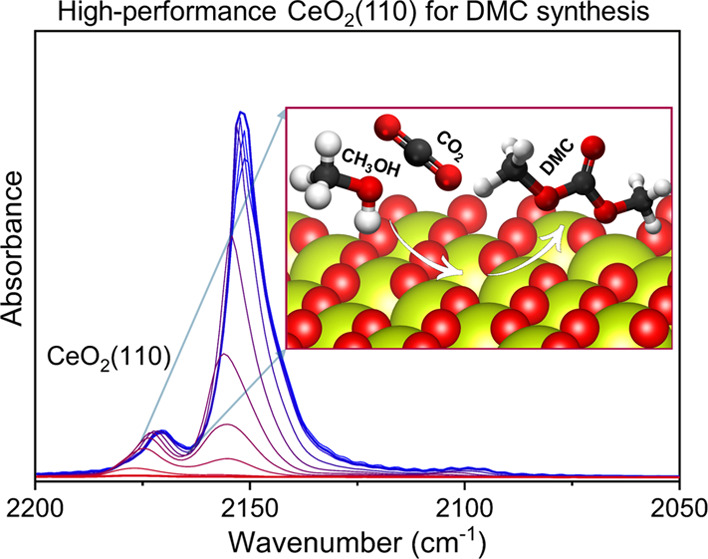

## 1 Introduction

With the advent of the Industrial Revolution, reducing atmospheric CO_2_ concentrations has become a major global challenge. One promising approach is its chemical fixation, which not only contributes to lowering CO_2_ levels but also transforms it into a valuable feedstock—particularly when facilitated by advanced heterogeneous catalysts. In this context, the direct carboxylation of alcohols using CO_2_ as a chemical feedstock provides a more environmentally friendly alternative to conventional technologies based on phosgene, which involve toxic reagents and costly raw materials.^1–3^

A notable example is dimethyl carbonate (DMC), produced through the direct reaction of methanol with CO_2_.^4,5^ Over recent decades, DMC has gained significant attention due to its wide range of applications, including its use as an electrolyte in lithium batteries, a fuel additive, a green solvent, and an intermediate in organic synthesis.^6–8^ For these reasons, the direct synthesis of DMC is considered one of the most suitable and eco-friendly routes for CO_2_ utilization.

However, this process still faces significant challenges. The high thermodynamic stability and kinetic inertness of the CO_2_ molecule hinder its activation and conversion. Moreover, side reactions such as catalyst degradation and carbonate hydrolysis—caused by the water generated during the reaction—can poison the catalyst surface and shift the reaction equilibrium back toward the reactants.^9,10^ Given these limitations and the requirements for large-scale applications, heterogeneous catalysis remains one of the most practical and promising approaches for the efficient conversion of CO_2_ into DMC.

CeO_2_-based materials are widely recognized as effective catalysts for DMC synthesis due to their acid-base characteristics and redox properties, particularly the presence of oxygen vacancies (Ce^3+^-V_o_) on their surfaces.^11,12^ These vacancies can enhance CO_2_ activation, serving as reactive sites and playing a key role in oxygen storage during oxidation reactions.^13–15^

However, the presence of oxygen vacancies does not always lead to improved DMC synthesis. Some studies report catalyst deactivation due to the reduction of Ce^4+^ to Ce^3+^,^16,17^ while others suggest that a higher concentration of Ce^3+^-V_o_ correlates with better catalytic performance.^18–20^ Although these findings appear contradictory, they can be reconciled by considering the locations of the surface oxygen vacancies. It is now understood that the location of these vacancies significantly influences the catalytic activity in DMC synthesis.

For example, Zhao et al.^21^ reported that while rod-shaped CeO_2_ exposes both (111) and (110) crystal planes, octahedral CeO_2_ exposes only the (111) plane. DFT calculations and experimental data demonstrated that methanol reactivity on the surface of CeO_2_ with exposed (110) planes is higher than that observed on (111) facets.^22^ As a result, the rod morphology exhibited significantly higher catalytic activity in comparison to the octahedral form. This suggests that the specific crystal planes exposed play a crucial role in the catalytic performance. Guan et al.^23^ further claimed that electron-rich Ce^3+^-V_o_ structures located on the (110) plane contribute to its excellent performance in DMC synthesis. More recently, Kang et al.^24^ reported that while Ce^3+^-V_o_ sites located on (111) planes inhibit DMC formation, those present on (110) planes promote it. In line with these findings, we recently reported the synthesis of a highly defective MOF-derived CeO_2_ catalyst, which also serves as an effective support for highly dispersed metallic species.^25,26^ This material contains coordinatively unsaturated Ce sites (CUS) predominantly located on the (110) planes, which act as active sites for DMC synthesis. Our study concluded that an optimal balance of Ce^3+^-V_o_ sites is critical for achieving high DMC yields.

Given that the role of Ce^3+^-V_o_ structures and surface reactivity in the direct synthesis of DMC remains inconclusive in the current literature, this work focuses on a comparative study of two different CeO_2_ catalysts. One is synthesized via a hydrothermal method starting from a Ce^4+^ precursor, resulting in a high surface area (HSA) oxide with potential Ce^3+^-V_o_ sites primarily located on (111) surface planes. The other is obtained by the calcination of a MOF precursor, yielding a highly defective cerium oxide with accessible (110) planes. These materials were thoroughly characterized by means of IR spectroscopy of adsorbed CO to study the accessibility of the centers and by *in situ* ambient pressure near-edge X-ray absorption spectroscopy (AP-NEXAFS) and IR spectroscopies to obtain fundamental insights into the catalysts’ surface properties and disclose the relation between reaction intermediates and Ce oxidation state. While CeO_2_-HSA produces a high proportion of surface HCOO-Ce^3+^ during methanol adsorption, which causes catalyst deactivation, CeO_2_-MOF presents a high concentration of CUS sites located on (110) planes, which are beneficial for the direct synthesis of DMC from CO_2_ and methanol.

## 2 Experimental Section

### 2.1 Catalyst Preparation

#### 2.1.1 MOF-Derived Synthesis of CeO_2_

The MOF-derived CeO_2_, named CeO_2_-MOF, was obtained by the calcination of the UiO-66 precursor. For that, UiO-66­(Ce) was initially prepared following a scaled-up previously reported procedure:^27^ 0.89 g of terephthalic acid (H_2_BDC) was dissolved in 30 mL of *N,N*-dimethylformamide (DMF). Subsequently, 10.0 mL of an aqueous solution of cerium­(IV) ammonium nitrate (0.53 M) was added. Then, the flask reactor was placed in an oil bath and heated to 100 °C for 15 min under magnetic stirring. The resulting pale-yellow solid was collected by centrifugation, washed with DMF and acetone, and finally dried in an oven at 80 °C. Afterward, the as-obtained UiO-66­(Ce) sample was calcined at 450 °C for 4 h at a heating ramp of 5 °C/min in air (0.5 mL/min) to form the corresponding CeO_2_-MOF oxide.

#### 2.1.2 Hydrothermal Synthesis of CeO_2_

The synthesis of CeO_2_ was modified from the literature^28,29^ and is based on the preparation of a ceria suspension from Ce­(IV) hydroxynitrate followed by thermal hydrolysis. For preparing ceric salt, 0.25 L of an aqueous solution of 0.37 N NH_3_ (30%) was added dropwise to 0.10 L of 0.98 M aqueous solution of cerium­(IV) ammonium nitrate until a pH of 0.5–0.8 was reached. The obtained colloidal suspension was then heated to 100 °C for 4 h to give a pale-yellow precipitate. The sample was recovered by centrifugation and calcined at 400 °C for 4–6 h to determine the Ce content (62–64% CeO_2_).

Subsequently, 9.3 g of the wet yellow solid was suspended in 0.12 mL of a 2 N NH_3_ solution. This suspension was added into a Teflon-lined autoclave and heated in an oven for 1 h at 180 °C. The as-obtained pale-yellow solid was then filtered and washed with a small amount of water. Finally, the material was dried and calcined at 350 °C for 6 h to obtain the corresponding oxide, denoted as CeO_2_-HSA.

### 2.2 Basic Characterization of the Synthesized Materials

Powder X-ray diffraction (PXRD) patterns were acquired with a PANalytical PW3050/60 X′Pert PRO MPD diffractometer equipped with a Cu anode (Kα = 1.5418 Å, Kβ removed by a Ni filter) and a X′Celerator detector using the Bragg-Brentano geometry. The crystallite size was estimated by FullProf software.^30^

Transmission electron microscopy (TEM) and high-resolution transmission electron microscopy (HR-TEM) were performed on a FEI Talos F200S electron microscope using an acceleration voltage of 200 kV. The analysis of HR-TEM images to identify interplanar distances was done with ImageJ software.

Specific surface area (SSA) measurements were performed at 77 K using a Micromeritics 3Flex physisorption analyzer. Before the measurements, the oxides were outgassed under vacuum at 400 °C for 1 h. SSA values were calculated by applying the Brunauer–Emmett–Teller (BET) equation to the collected N_2_ adsorption/desorption isotherms.

Fourier transform IR (FT-IR) spectroscopy in transmission mode was used to investigate the surface defects of the materials by monitoring the desorption of CO as a probe molecule. The corresponding CeO_2_ material was pressed in a self-supported pellet of area ≈ 10 cm^2^/g using a hydraulic press (5 ton/cm^2^). The pellet was placed in a gold holder and inserted into a quartz IR cell, designed to allow thermal treatments under controlled atmospheres and to collect spectra at liquid nitrogen temperature (LNT). Before analysis, the sample was pretreated as follows: it was degassed at 400 °C (heating ramp of 5 °C/min) until the pressure reached 5 × 10^–4^ mbar. Afterward, O_2_ (100 mbar) was introduced into the cell and kept at this temperature for 30 min, with the gas being refreshed every 10 min. The system was then cooled to RT and evacuated again to 5 × 10^–4^ mbar. Without the sample being exposed to air, the cell was connected to a dedicated gas line. The prepared cell was mounted on a Bruker Vertex 70 spectrophotometer equipped with a mercury cadmium telluride (MCT) cryo-detector, operating in the 4000–600 cm^–1^ spectral range with 2 cm^–1^ resolution. A dose of 35 mbar of CO was introduced, and liquid nitrogen was added to the reservoir to cool the system to cryogenic temperature (LNT). Once thermal equilibrium was achieved, as inferred by the stabilization of the CO signal, the spectrum of the maximum CO coverage was measured. Finally, the CO was gradually evacuated by controlled volume expansion until complete removal.

### 2.3 Ambient Pressure Near-Edge X-ray Absorption Spectra (AP-NEXAFS) Study

AP-NEXAFS measurements were performed at the APE-HE beamline of the Elettra Italian Synchrotron radiation source. CeO_2_ was held in a homemade reactor cell, which allows thermal treatments in the 25–400 °C range under atmospheric pressure.^31^ The NEXAFS spectra at Ce M_4,5_-edges were collected with TEY mode in the 870–910 eV range and 0.01 eV energy resolution. The measurement protocol is reported in Figure S1. Spectra have been background subtracted, normalized by the area of the first peak (M_5_-edge), and energy aligned to the first spectra of the series using the OriginLab (OriginPro 2018) software. Ce^4+^/Ce^3+^ spectral pure components and their concentration evolution were extracted using both linear combination fit (LCF) and multivariate curve resolution-alternated least squares (MCR-ALS).^32^ The LCF analysis was performed with the ATHENA code from the Demeter suite,^33^ using the spectra of commercial CeO_2_ and CeF_3_ (measured on the same beamline) as reference for pure Ce^4+^ and Ce^3+^ species, respectively. MCR-ALS was performed using the MATLAB-based GUI by Jaumot and co-workers,^34^ where spectra and concentration were constrained to positive values while the closure condition was applied to concentrations. CeO_2_ and CeF_3_ spectra were employed as references of Ce^4+^ and Ce^3+^ components, respectively, and subsequently added at the end of the data set to help the MCR-ALS protocol in finding these pure spectral components.

### 2.4 *In Situ* IR Study

*In situ* FT-IR spectra were acquired in transmission mode by using an Aabspec cell, which allows thermal treatments under atmospheric pressure. The cell was connected to a Bruker Invenio R spectrophotometer. Spectra were acquired in the 4000–500 cm^–1^ range at a resolution of 2 cm^–1^. In a typical experiment (Figure S2), the CeO_2_ surface was previously cleaned from adsorbed species (*i.e.*, H_2_O, carbonates, and bicarbonates) by flowing (40 mL/min) a mixture of N_2_:O_2_ (1:1) at 400 °C during 30 min with a heating ramp of 5 °C/min. The temperature was then held at 400 °C for 30 min and then lowered to RT. Then, the temperature was lowered, and to prevent self-reduction during cooling, the aerobic atmosphere was maintained until 150 °C, while from that moment the gas stream consisted of pure N_2_ (40 mL/min). Once the reaction temperature was reached, 38 mL/min of pure N_2_ was passed through a saturator containing methanol, so that a mixture of N_2_ and methanol reached the sample. Spectra were acquired every 20 s until two consecutive collected spectra were identical. Then, the methanol flux was stopped, and a mixture of CO_2_ (2 mL/min) and N_2_ (38 mL/min) was dosed into the cell (spectra were acquired every 20 s until an equilibrium was reached). The same experiment was performed on another fresh pellet of the same sample, sending a mixture of CO_2_ (2 mL/min) and N_2_ (38 mL/min) to monitor the adsorption and/or formation of the adsorbed CO_2_ intermediates.

### 2.5 Catalytic Studies

The catalytic tests were carried out in a Teflon-lined stainless autoclave equipped with a magnetic stirrer, heating plate, and thermocouple. First, ∼80 mg of catalyst was placed in the reactor and activated at 200 °C for 1 h under O_2_ flux (10 mol % in He, ∼50 mL/min). Then, the reactor was cooled to RT by using an ice bath. After the activation, a solution of anhydrous methanol (6.40 g, 200 mmol) and 1-propanol (35 mg, 0.58 mmol) was placed in the reactor. Since the reaction is highly sensitive to the presence of water, which poisons the catalyst and pushes the reaction equilibrium toward the reactants, the methanol used for the catalytic test was anhydrous, and a Schlenk line was used for the withdrawal of water. The 1-propanol acts as an internal standard for the quantification of DMC produced. The magnetic stirrer was then added, and the autoclave was closed. Then, the reactor was pressurized with pure CO_2_ up to 30 bar. The reaction mixture was heated under magnetic stirring at 140 °C for 3 h (pressure raised to ∼52 bar). Finally, the reaction was quenched with an ice bath and analyzed with a gas chromatograph equipped with a FID and TCD detectors. The DMC yield was calculated and normalized by the BET SSA of the catalyst (*Y*_s_, mmol_DMC_ m^–2^).

## 3 Results and Discussion

### 3.1 Structure and Morphology of the Materials

In this work, we prepared two CeO_2_ catalysts by following two different synthetic approaches: the first one is based on a hydrothermal method to obtain a high surface area oxide (surface area >300 m^2^/g after calcination at 350 °C for 6 h), named as CeO_2_-HSA. The second is obtained by calcining a Ce-MOF to yield a highly defective oxide, denoted as CeO_2_-MOF. These materials are first characterized by PXRD, showing in both cases, diffraction patterns typical of face-centered cubic (*fcc*) cerium oxide, since their Bragg peaks could be indexed as the (111), (200), (220), (311), (222), (400), (331), and (420) planes (see Supporting Information, Figure S3).^35^ We could observe broader and weaker diffraction peaks for the CeO_2_-HSA with respect to the MOF-derived sample, indicating a smaller crystallite size. Moreover, CeO_2_-HSA showed a broad contribution at a lower diffraction angle, which could be associated with a minor amorphous fraction.^36^ Figure S4 presents transmission electron microscopy (TEM) and high-resolution TEM (HR-TEM) images of the as-prepared CeO_2_-HSA and CeO_2_-MOF samples. In the HR-TEM image of CeO_2_-HSA, distinct lattice fringes corresponding to the (111) plane are observed, with an interplanar spacing of 0.31 nm, indicating that CeO_2_-HSA predominantly exposes the (111) surface. In contrast, the HR-TEM image of CeO_2_-MOF reveals two sets of lattice fringes, assigned to the (111) and (220) planes, with interplanar spacings of 0.31 and 0.19 nm, respectively.^37,38^ Finally, BET values are in agreement with the observations from PXRD analysis since CeO_2_-HSA, composed of smaller crystallites, possesses a higher surface area (304 m^2^ g^–1^ for CeO_2_-HSA, 29 m^2^ g^–1^ for CeO_2_-MOF).

Fourier transform IR spectroscopy in transmission mode was used to study the nature and accessibility of the surface sites and their defectivity by following the chemisorption of CO as a probe molecule that causes a shift in the IR signal with respect to the CO gas phase. At maximum CO coverage, we detected a main contribution centered at 2151 cm^–1^ in the IR spectra that could be related to CO linearly adsorbed on the Ce sites of the (111) plane, including both van der Waals interactions and those between the CO molecule and Ce–OH groups (Figure 1).^39–41^

**Figure 1:**
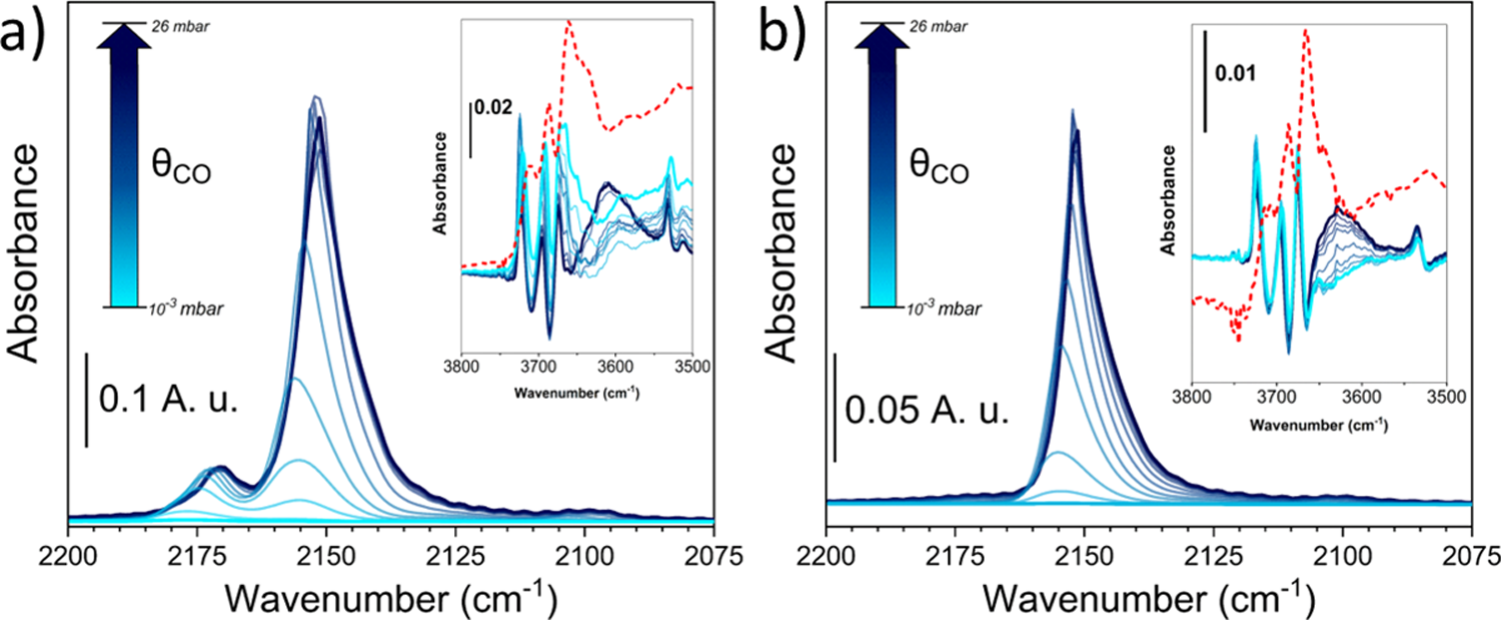
Difference IR spectra of CO desorption (from blue to light blue line) at LNT as a function of the CO coverage (θ_CO_) on (a) CeO_2_-MOF and (b) CeO_2_-HSA. *v*(OH) regions are reported in the insets with the spectra of the activated sample at RT reported in the red-dashed line, without any subtraction.

However, a less intense feature at ∼2170 cm^–1^ was only observed in the case of the CeO_2_-MOF sample. This component at higher wavenumbers could be ascribed to the interaction of CO with coordinatively unsaturated (CUS) Ce sites on the (110) plane, whose presence was demonstrated by TEM analysis, and its shift toward higher wavenumbers with respect to the gas phase value (2143 cm^–1^) is an indication of its Lewis acid strength.^42,43^

By decreasing the CO coverage, it was possible to observe a red shift of ∼5 cm^–1^. This shift is due to the reduction of the “through space” (related to dipole–dipole coupling between parallel vibrating molecules) and “through solid” (associated with vibrational coupling across binding electrons) interactions that played a major role at high coverage.^44–46^ Furthermore, we could observe that during the desorption, the intensity of the band associated with CO linearly adsorbed on nondefective Ce^4+^ sites rapidly decreased, while the one related to CO on defective sites persisted, meaning that those sites on CeO_2_-MOF are more energetic.

### 3.2 Study of the Ce Oxidation State by *In Situ* AP-NEXAFS Spectroscopy

AP-NEXAFS spectroscopy was employed to unravel the Ce oxidation state on CeO_2_ samples during the adsorption of the CO_2_ and CH_3_OH molecules. The protocol is described in the Experimental Section, while the Ce M_5_-edge spectra obtained in each step are reported in Figure S5. The materials were already in their oxidized state at RT, and no changes were observed during the thermal activation. After the activation, the samples were exposed to a CO_2_ atmosphere as described in Figure S1. However, as expected, the Ce oxidation state did not change during its exposure to CO_2_ (Figure S6). In another set of experiments, we studied the methanol effect on the electronic properties of Ce. After exposing the samples to methanol, two-band shoulders appeared at lower energies (880.7 and 879.6 eV), which indicated a partial reduction of Ce, induced by methoxide-to-formate decomposition (see Figure 2a,c).^47,48^ However, the cerium oxidation state did not change after CO_2_ coadsorption, indicating that the formed Ce^3+^ sites during methanol adsorption persisted during the whole reaction (Figure S5).

**Figure 2:**
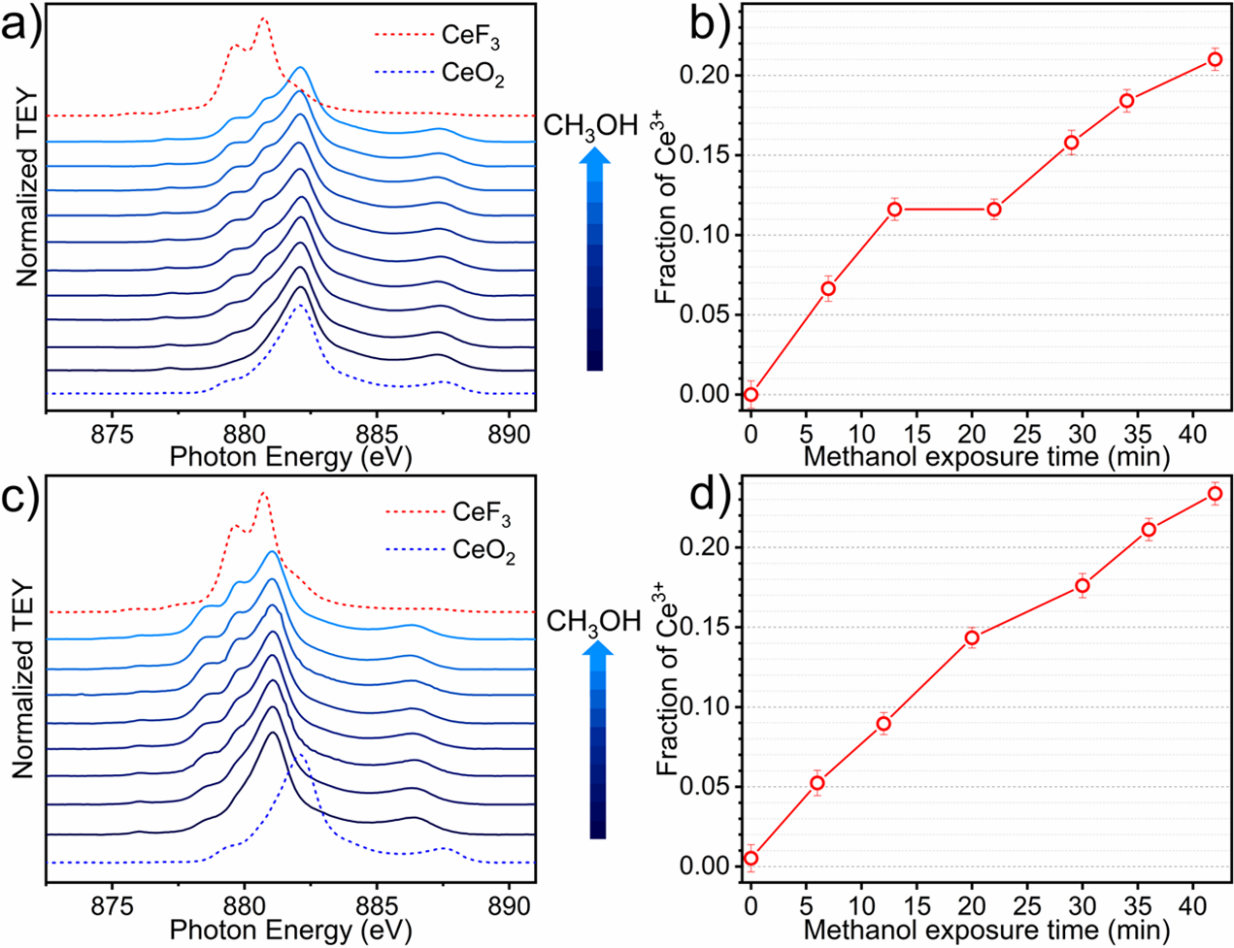
(a) CeO_2_-MOF and (c) CeO_2_-HSA Ce M_5_-edge *in situ* AP-NEXAFS spectra obtained during methanol adsorption (from blue to light blue line). Ce^3+^ concentration profile evolution during methanol adsorption on (b) CeO_2_-MOF and (d) CeO_2_-HSA derived from MCR-ALS analysis applied to the experimental data in panels (a) and (c). CeF_3_ and CeO_2_ references are shown as red and blue dotted line spectra, respectively.

The concentration evolution of Ce^3+^/Ce^4+^ species was extracted by using both LCF and MCR-ALS, showing in both cases a similar trend with a minor offset in the absolute concentration values (Figure S7). CeO_2_ and CeF_3_ spectra were employed as the Ce^4+^ and Ce^3+^ references, respectively. From the LCF analysis, we could observe that both samples were already close to being fully oxidized at RT (ca. 100% Ce^4+^) and remained oxidized during activation (Figure S5). Then, when methanol was flowed into the chamber, the concentration of Ce^4+^ decreased, while we achieved 20 and 26% of Ce^3+^ species for CeO_2_-MOF and CeO_2_-HSA, respectively. Even by sending CO_2_, the samples could not be fully oxidized, being the Ce^3+^ concentration practically unchanged. On the other hand, the MCR procedure identified two principal components describing 99.6% of the variance. In this case, we could observe a similar trend but with bigger oscillations. For example, we could observe a more pronounced reduction when methanol was in the gas feed, where we reached ∼30% of Ce^3+^. The quantitative results obtained by both LCF and MCR-ALS are in complete agreement with the considerations made in the qualitative analysis of the spectra, confirming that the formation of the two-band shoulders at lower energies when methanol was in the gas feed could be related to the reduction of the Ce^4+^ into Ce^3+^.

### 3.3 Evolution of Surface Species Followed by *In Situ* IR Spectroscopy

*In situ* IR spectroscopy was employed to monitor the evolution of the different intermediates/products on the catalyst surface during the reaction between CO_2_ and methanol. The sample was first activated by following the procedure described in the experimental section with the objective of cleaning the catalyst surface from adsorbed species (*i.e.*, H_2_O, carbonates, and bicarbonates). FT-IR spectroscopy was used to monitor methanol and CO_2_ adsorption on the catalyst surface at reaction temperature (150 °C) and to follow the formation of the intermediates/products on the catalyst surface.

#### 3.3.1 Methanol Adsorption

Methanol can interact with the surface of CeO_2_ catalysts by the oxygen atom, forming on-top (type I), bridging (type II), and/or three-coordinated (type III) methoxy species (Ce–OCH_3_). The spectral evolution of methanol adsorption over CeO_2_-MOF and CeO_2_-HSA is reported in Figure 3a,b, respectively, showing that gas phase methanol, recognized by its representative P, Q, and R branches, is quickly adsorbed on the catalyst surface. Parallelly, bands associated with type I and type II methoxy are clearly observed in the 1050–1110 cm^–1^ range.^49^ The blue shift in the ν­(CO) signal of type I methoxy groups from CeO_2_-MOF (1098 cm^–1^) to CeO_2_-HSA (1103 cm^–1^) indicates that these species are located on both (110) and (111) planes in the MOF-derived sample, whereas they reside mainly on the (111) facets in CeO_2_-HSA.^50^ Bending and stretching modes for these intermediates can also be observed in the 1430–1450 and 2800–2950 cm^–1^ ranges (see assignment in Table S1).^51,52^ Parallel to methoxy species formation, we noticed four intense signals (1371, 1380, 1552, and 1576 cm^–1^) associated with formate stretching and bending modes.^43,53^ We could assign those bands to b-HCOO^–^ formed on Ce^3+^ (1380 and 1576 cm^–1^) and on Ce^4+^ (1371 and 1552 cm^–1^) sites.^54^ Interestingly, the relative proportion of HCOO-Ce^3+^ is higher in the case of CeO_2_-HSA with respect to that of the CeO_2_-MOF sample. This could have a negative effect on catalysis since we demonstrated in a previous work that the saturation of HCOO/Ce^3+^ species on the catalyst surface causes the material deactivation.^26^

**Figure 3:**
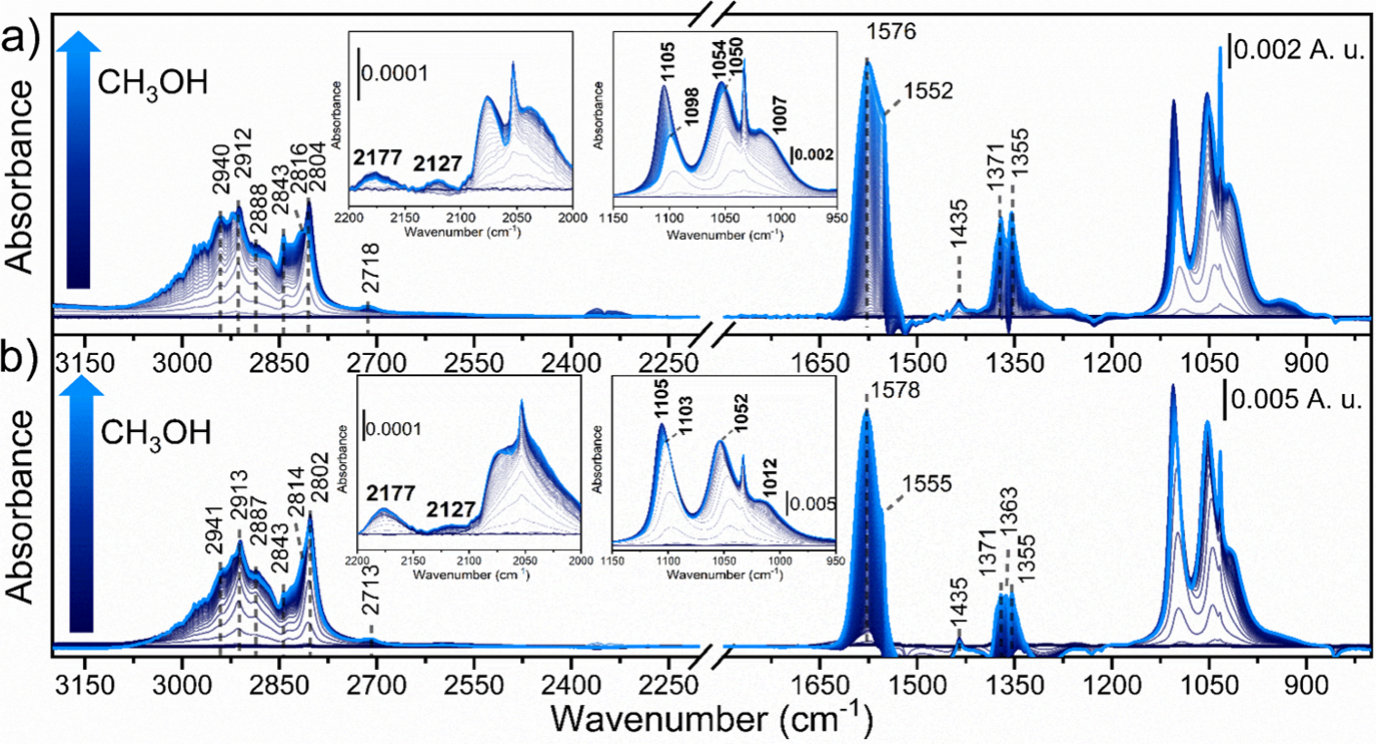
*In situ* difference IR spectra collected during methanol adsorption (from blue to light blue line) at 150 °C over (a) CeO_2_-MOF and (b) CeO_2_-HSA. Details of the Ce^3+^ transitions are reported in the inset.

Furthermore, we could observe the Ce^3+^ fingerprint at 2127 cm^–1^ in both cases,^55–57^ meaning that methanol was able to reduce the catalyst surface, confirming the results of the quantitative analysis of Ce-speciation from M_5_-edge NEXAFS. Since IR and NEXAFS studies indicated an overall Ce^4+^ to Ce^3+^ reduction when methanol is adsorbed at 150 °C, we can hypothesize that methanol interacts with the catalyst surface to form methoxy species (Ce–OCH_3_), which can be further transformed into formate species (Ce–HCOO), causing the cerium reduction.

#### 3.3.2 CO_2_ Adsorption

CO_2_ may act as a Lewis acid, forming carbonate species, or a Lewis base toward residual OH groups, forming bicarbonates. The spectral evolution of the adsorption of CO_2_ over CeO_2_-MOF and CeO_2_-HSA is reported in Figure 4a,b, respectively. The adsorption of CO_2_ into the catalyst surface results in the formation of carbonates and bicarbonates, recognized by the representative ν­(COO) and δ­(OH) vibrations (see assignments in Table S2).^58–60^ Moreover, the signals at 1216 and 1392 cm^–1^ could be assigned to the formation of two bicarbonates h-CO_3_^–^ species (I, characterized by slightly higher frequencies, and II, that may result from the adsorption of CO_2_ into surface OH groups either mono- or bidentate). This could be confirmed by looking at the *v*(OH) region (insets in Figure 4). Hydroxyl groups are consumed, while h-CO_3_^–^ can be identified from their characteristic bands at 3618 cm^–1^.

**Figure 4:**
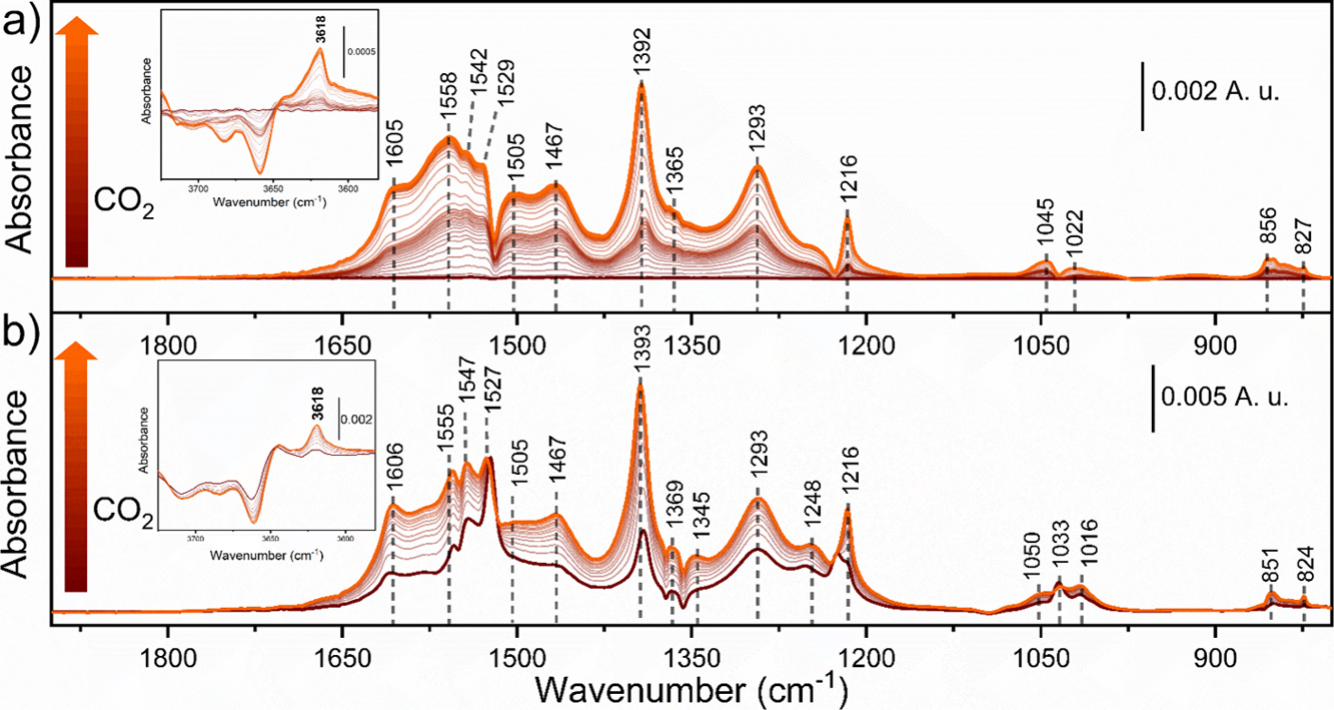
*In situ* difference IR spectra collected during CO_2_ adsorption (from dark red to orange line) at 150 °C over (a) CeO_2_-MOF and (b) CeO_2_-HSA. Details of OH stretching vibrations are reported in the inset.

#### 3.3.3 CO_2_ Coadsorption

Figure 5 displays the spectral evolution during the sequential adsorption of methanol and CO_2_. For both samples, we could observe the consumption of the methoxy species (identified by the bands at ∼1100, ∼1050, and 2177 cm^–1^ (methoxy overtone) and the ones in the 2800–2950 cm^–1^ range). At the same time, we could observe the formation of new features related to different types of carbonates. The bands related to formates were conserved (∼2718, ∼2846, ∼2930, ∼1370, and ∼1570 cm^–1^), meaning that only methoxy species react efficiently with CO_2_ to form monomethyl carbonate (MMC) intermediate.^26^ The characteristic bands of the MMC intermediate were not clearly observed, since their signals (∼1360, ∼1470, and ∼1600 cm^–1^) could be convoluted with those of formates (again, only a weak feature at ∼1470 cm^–1^ was observed).^49^ A new contribution at ∼1296 cm^–1^ was also observed and could be assigned to carbomethoxy intermediate.^61^ DMC was not detected, suggesting its thermal decomposition on the CeO_2_ surface under the studied conditions. Focusing on the Ce^3+^ fingerprint at 2127 cm^–1^, we could observe that this feature was consumed during CO_2_ coadsorption, in line with Ce^3+^ oxidation. Since we did not observe Ce oxidation during the NEXAFS experiment at comparable conditions (Figure S5), one possibility is that methoxy species adsorbed on the surface gave a methoxy-to-methyl decomposition, with the consumption of the Ce^3+^-V_o_ formed during methanol adsorption and the production of methyl species, fundamental for the MMC methylation.

**Figure 5:**
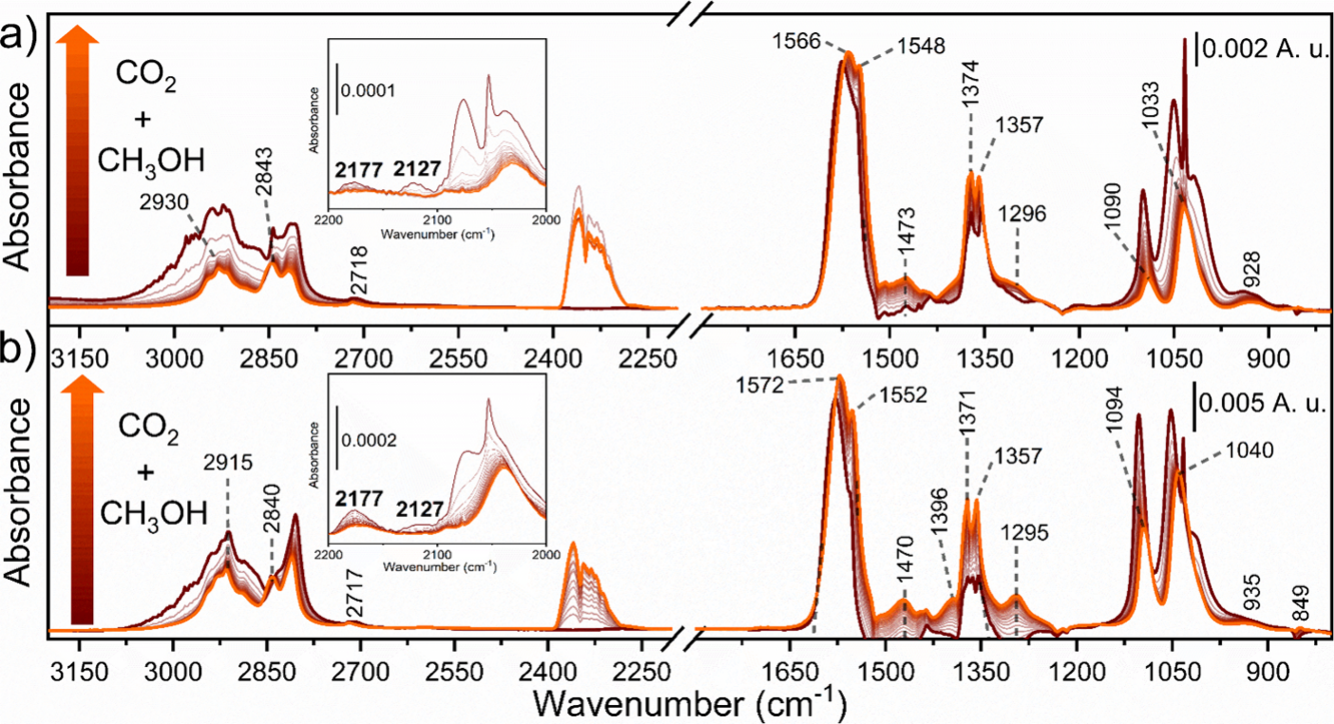
*In situ* difference IR spectra collected during CO_2_ coadsorption (from dark red to orange line) at 150 °C over (a) CeO_2_-MOF and (b) CeO_2_-HSA. Details of the Ce^3+^ transitions are reported in the inset.

### 3.4 Synthesis of DMC from CO_2_ and Methanol

Figure 6 shows the catalytic activity of the two CeO_2_ catalysts for DMC synthesis from the direct reaction of CO_2_ and methanol. Both materials presented similar DMC yields if compared to the literature results in the absence of a dehydrating agent (∼2.2 mmol/g_CAT_).^62–65^ However, normalization of the DMC yield by dividing by the surface area of the catalysts (Figure 6) shows a lower productivity for CeO_2_-HSA. This could be explained by the formation of a higher amount of surface HCOO-Ce^3+^ sites in the latter, which causes a catalyst deactivation, while CeO_2_-MOF presents a high concentration of CUS sites located on (110) planes, which are beneficial for the direct synthesis of DMC from CO_2_ and methanol.

**Figure 6:**
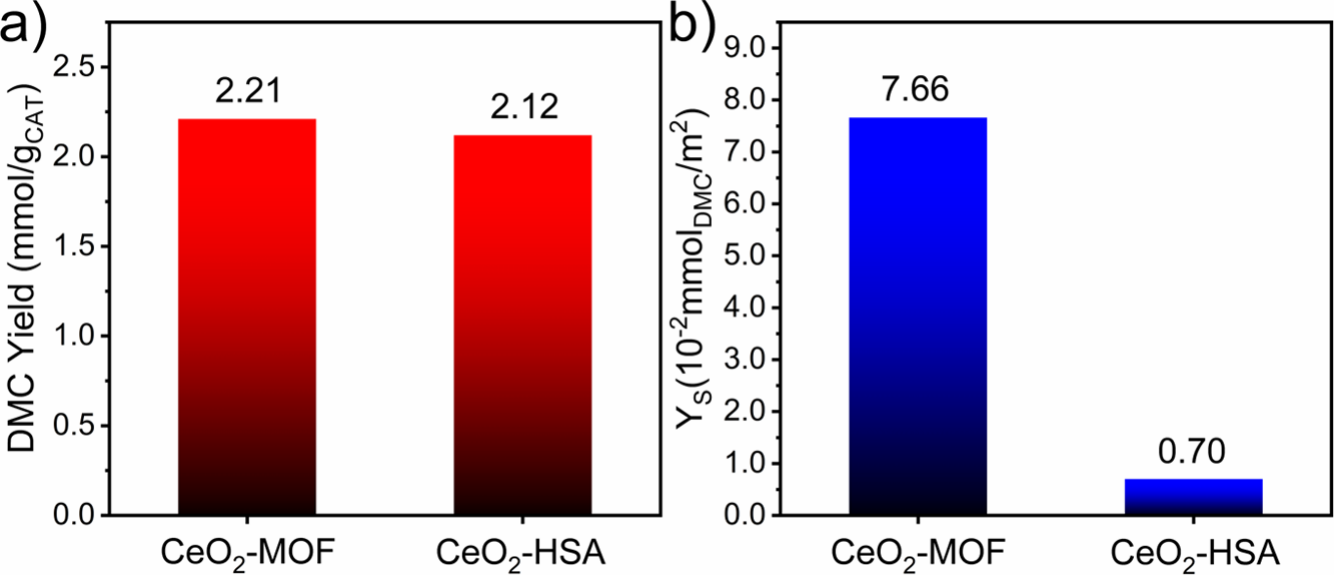
Normalized DMC yield by (a) the mass of catalysts and (b) the BET SSA.

## 4 Conclusions

In this study, we investigated the surface properties of two distinct CeO_2_ catalysts: one synthesized using a modified hydrothermal method (CeO_2_-HSA), and the other derived from the calcination of a metal–organic framework (MOF), referred to as CeO_2_-MOF. The hydrothermally synthesized CeO_2_-HSA exhibited a high surface area (surface area > 300 m^2^/g after calcination at 350 °C for 6 h) with accessible cerium sites predominantly exposed on the (111) crystal planes. In contrast, CeO_2_-MOF featured a significant presence of coordinatively unsaturated cerium sites (CUS) located mainly on the (110) planes.

To elucidate the nature of surface intermediates and the oxidation state of cerium during the reaction, we employed *in situ* infrared (IR) and near-edge X-ray absorption fine structure (NEXAFS) spectroscopy. Both catalysts exhibited cerium reduction upon methanol adsorption, attributed to the transformation of methoxide species into formate. Notably, CeO_2_-HSA generated a higher proportion of surface-bound HCOO-Ce^3+^ species, which correlates with its larger surface area.

However, as previously reported, the increased population of surface HCOO-Ce^3+^ species in CeO_2_-HSA leads to catalyst deactivation over time. In contrast, CeO_2_-MOF, with its abundance of CUS sites on the (110) planes, demonstrated enhanced performance for the direct synthesis of dimethyl carbonate (DMC) from CO_2_ and methanol, highlighting the beneficial role of its unique surface structure.

## Supplementary Material



## Notes

Measurement protocols for in situ NEXAFS and FT-IR experiments, PXRD patterns and TEM/HR-TEM images of the materials, NEXAFS spectra and their corresponding concentration profiles obtained from MCR-ALS and LCF protocols, IR bands frequencies assignment for CO_2_, and methanol adsorption (PDF)
