# Supplementary material for: Understanding Surface Properties in CeO2 Catalysts for the Synthesis of Dimethyl Carbonate: A Combined In Situ IR and NEXAFS Study
Source: J Phys Chem C Nanomater Interfaces. 2025 Oct 23;129(44):19794–802. doi: 10.1021/acs.jpcc.5c04979 (PMC12599007; doi:10.1021/acs.jpcc.5c04979)
Supplement: Supplementary file 1 [file jp5c04979_si_001.pdf]

Understanding Surface Properties in CeO_2 Catalysts for the Synthesis of Dimethyl Carbonate: A Combined *In Situ* IR and NEXAFS Study

Gionata Galliano[a], Edoardo Bracciotti[a], Andrea Jouve[a], Luca Braglia[b,c], Rudy Calligaro[d], Elisa Borfecchia[a], Sergio Rojas-Buzo[a,e*], Silvia Bordiga[a]

[a] Department of Chemistry, NIS Center and INSTM Reference Center, University of Turin, 10125 Turin, Italy.

[b] Area Science Park, Padriciano 99, 34149, Trieste, Italy

[c] CNR-IOM, Strada Statale 14 Km 163.5, 34149 Basovizza, Trieste, Italy

[d] Dipartimento Politecnico, Università degli Studi di Udine, Via del Cotonificio 108, Udine, 33100 Italy

[e] IIQ, Instituto de Investigaciones Químicas (CSIC-Universidad de Sevilla), Avda. Americo Vespucio 49, Seville 41092, Spain.

[*] Sergio Rojas-Buzo: sergio.rojas@iiq.csic.es.

Table of contents

1. General materials characterization .... B

2. AP-NEXAFS spectroscopy .... D

3. *In situ* FT-IR spectroscopy .... G

4. References I

1. General materials characterization

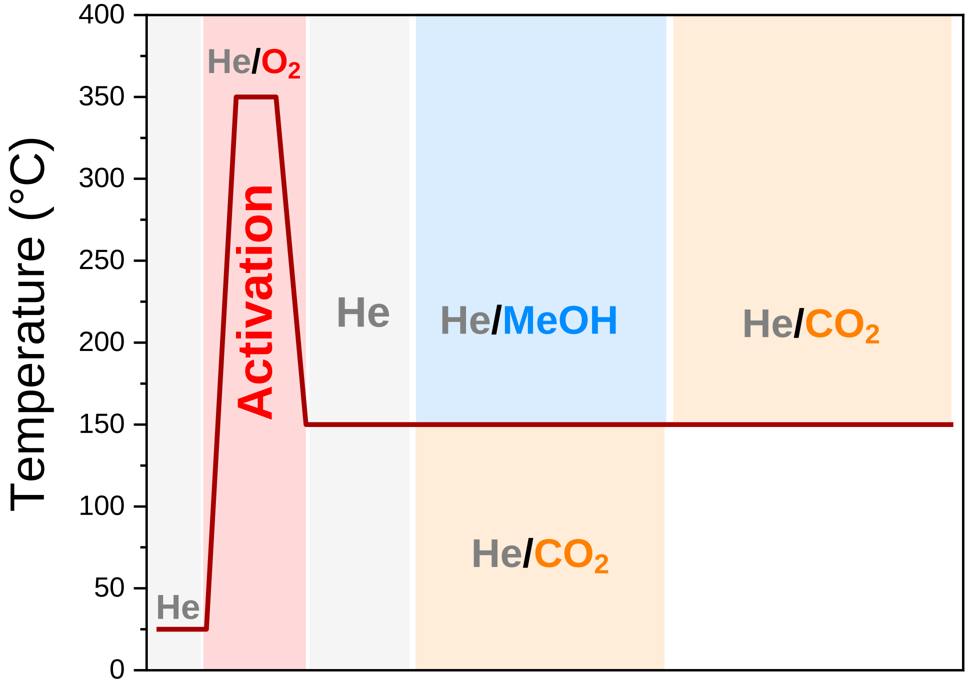

Figure S1. Measurement protocol used for the *in situ* NEXAFS experiments

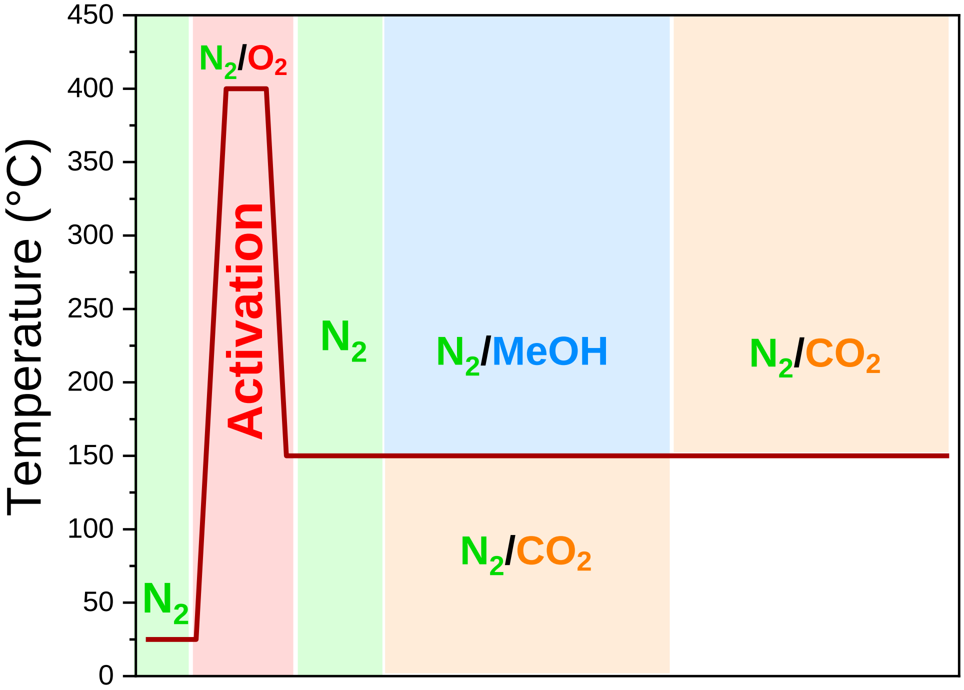

Figure S2. Measurement protocol used for the *in situ* FT-IR experiments

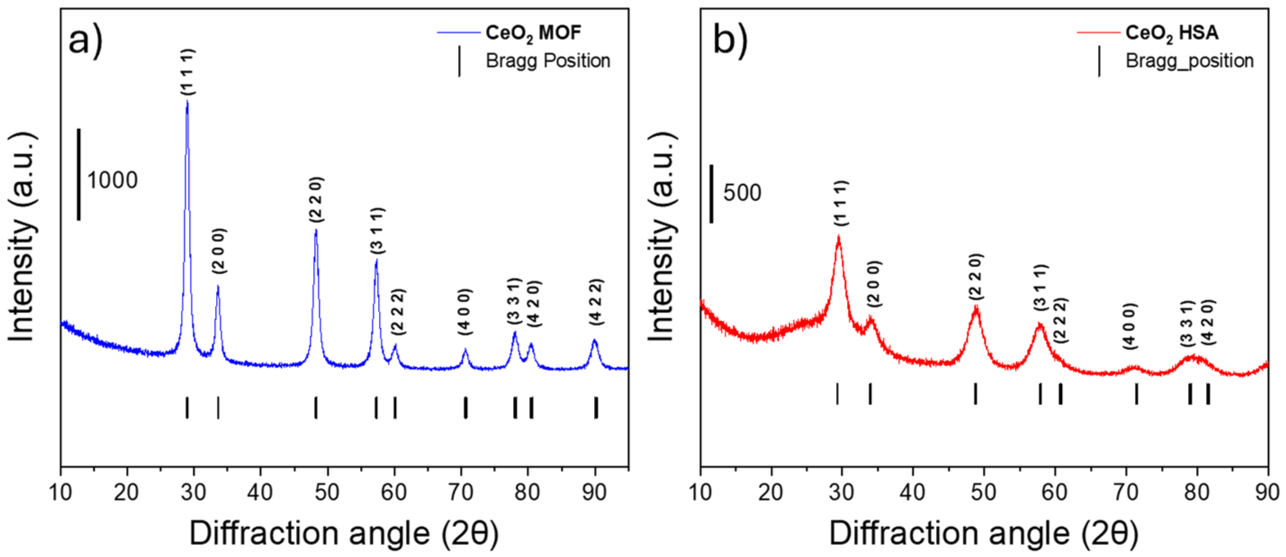

Figure S3. PXRD patterns of a) CeO_2-MOF and b) CeO_2-HSA. Black ticks represent Bragg positions typical of face-centered cubic (*fcc*) cerium oxide.

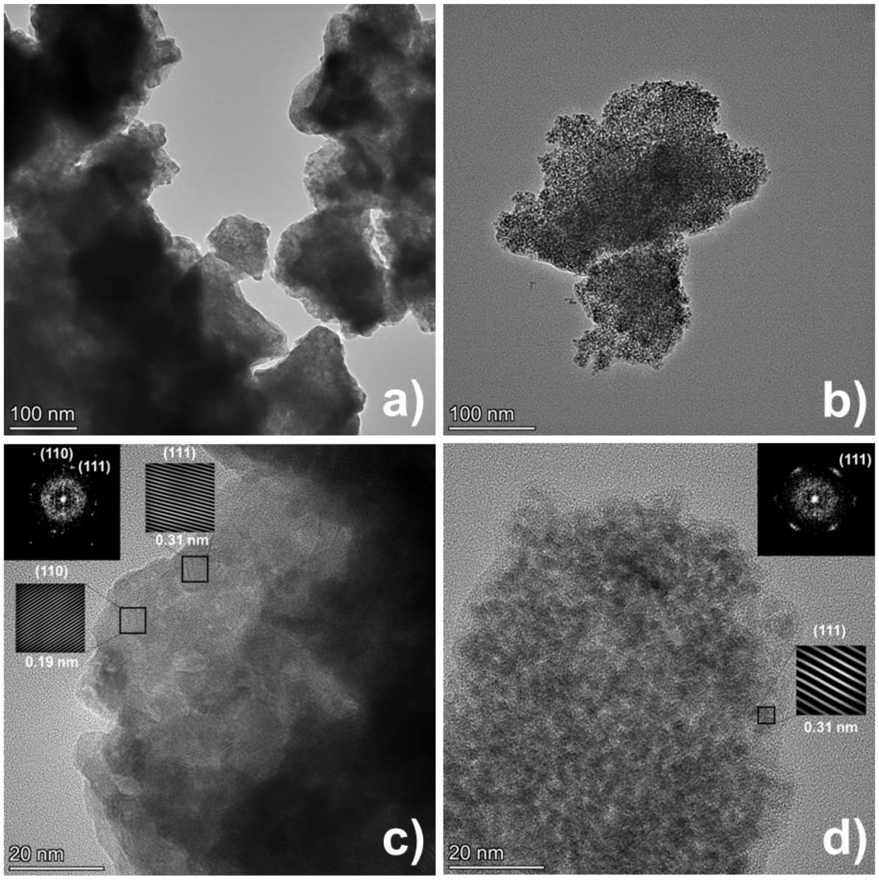

Figure S4. TEM and HR-TEM images of a) and c) CeO_2-MOF and b) and d) CeO_2-HAS, respectively.

2. AP-NEXAFS spectroscopy

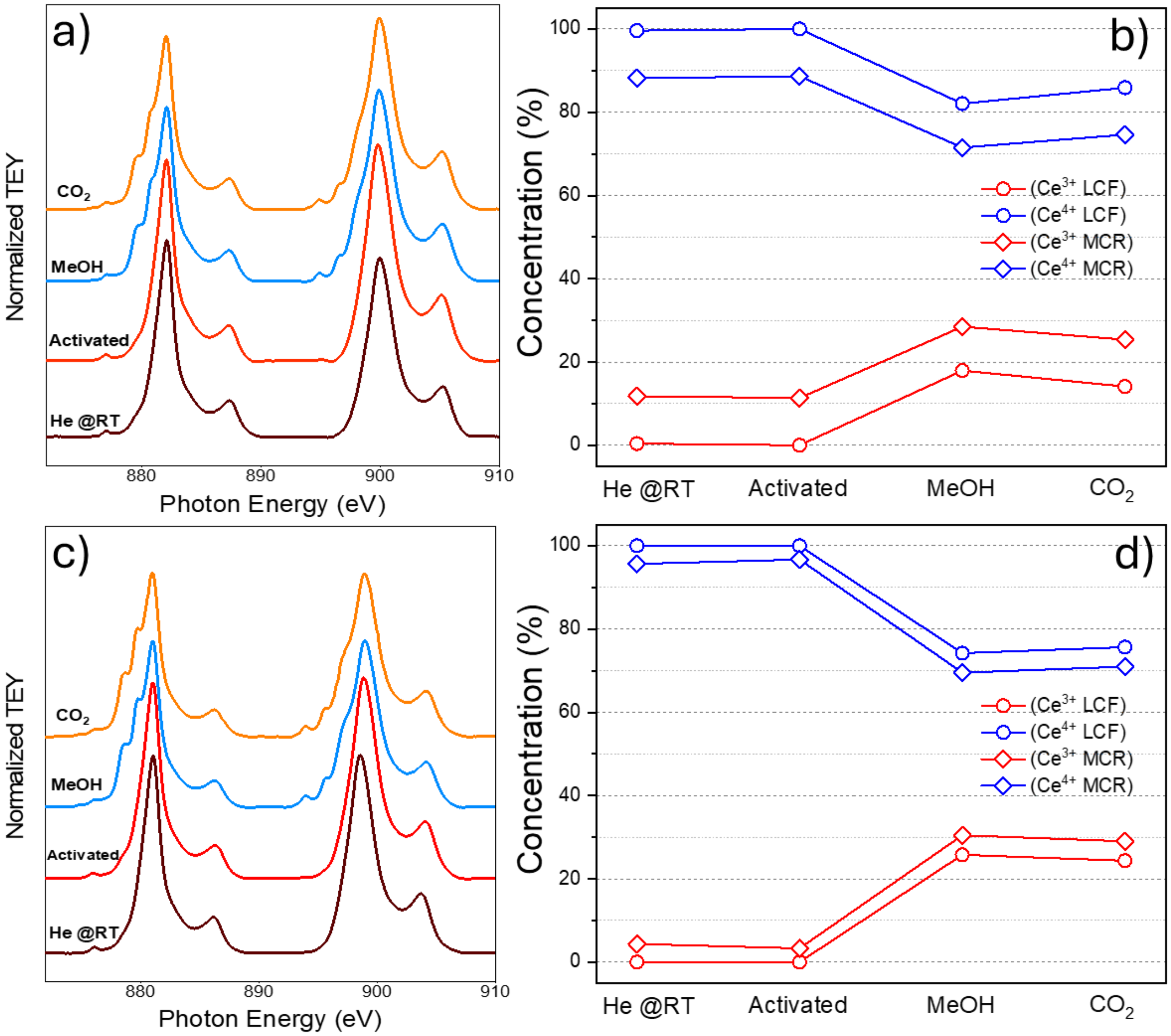

Figure S5. a) CeO_2-MOF and c) CeO_2-HSA Ce M_5-edge *in situ* AP-NEXAFS spectra measured at RT (brown), after activation (red), after MeOH (light blue) and after CO_2 co-adsorption (orange). b) CeO_2-MOF and d) CeO_2-HSA Ce^{4+}/Ce^{3+} concentration profile evolution obtained from LCF and MCR-ALS analysis applied to the experimental spectra in panels a) and c).

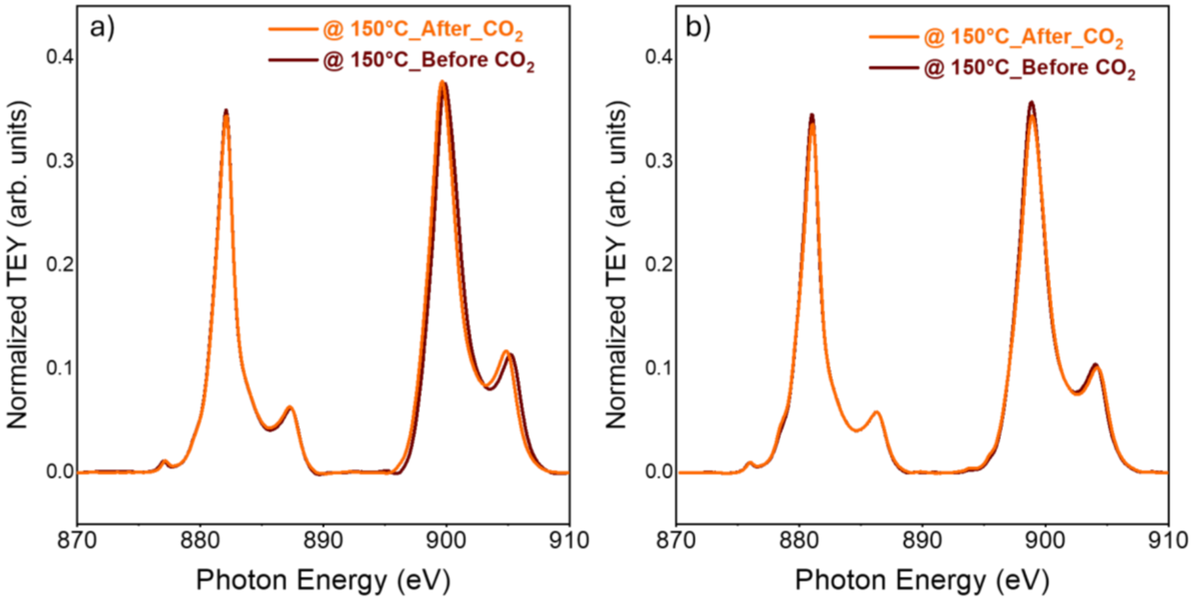

Figure S6. a) CeO_2-MOF and b) CeO_2-HSA Ce M_5-edge *in situ* AP-NEXAFS spectra measured after activation (brown) and after CO_2 adsorption (orange).

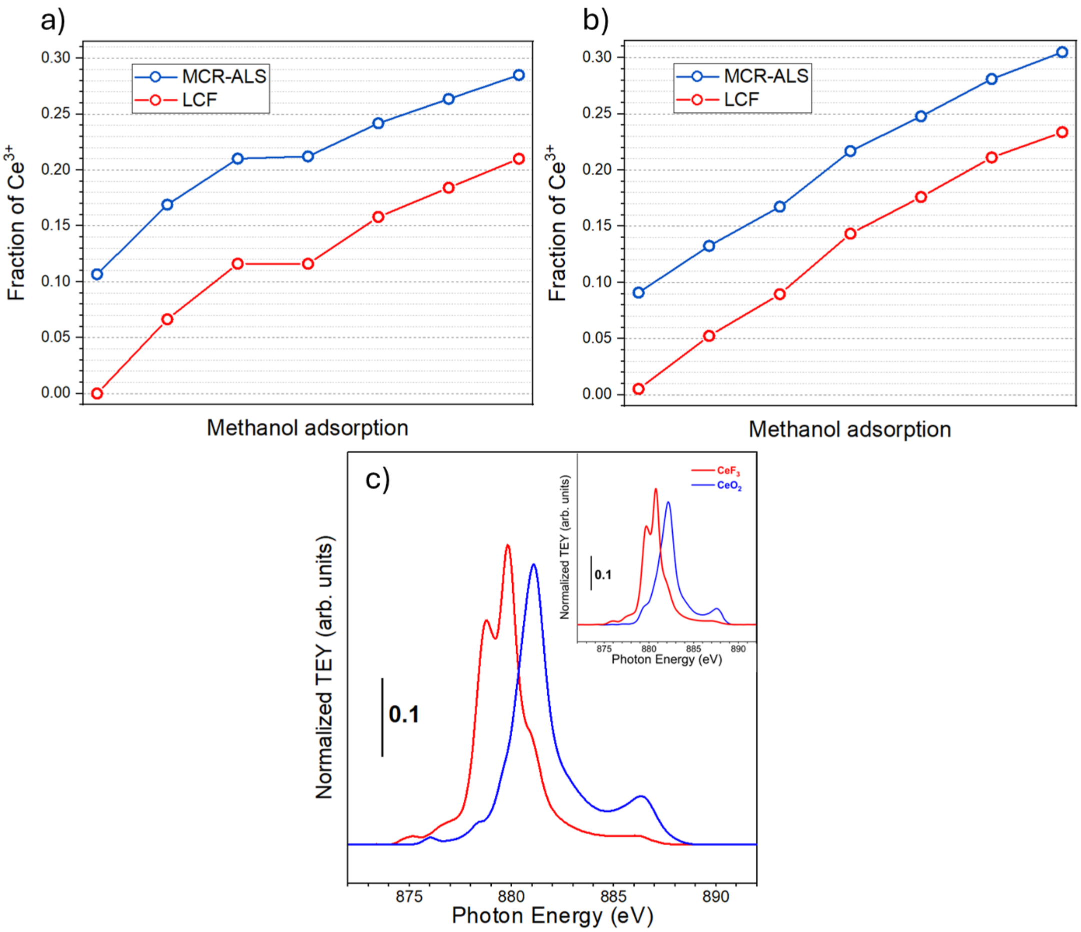

Figure S7. a) CeO_2-MOF and b) CeO_2-HSA Ce^{3+} concentration profile evolution during MeOH adsorption obtained from MCR-ALS (blue) and LCF (red) protocols. c) spectral component extracted from MCR-ALS (CeF_3 (Ce^{3+}) and CeO_2 (Ce^{4+}) reference spectra are reported in the top inset with red and blue lines, respectively).

3. *In situ* FT-IR spectroscopy

Table S1. Bands frequencies assignment obtained during methanol adsorption over CeO_2-MOF and CeO_2-HSA[1–4]

Frequency (cm^{-1})	Assignment	Species
1098-1110	v_{sym}(CO)	CH_3O-Ce (I)
1050-1061	v_{sym}(CO)	CH_3O-Ce (II)
1355-1366	v_{sym}(OCO)	$HCOO^-/Ce^{4+}$- Ce^{3+}
1371-1380	δ(CH)	$HCOO^-/Ce^{4+}$- Ce^{3+}
1435-1436	δ(CH)	CH_3O-Ce (I)
1446-1455	$\delta(CH_3)$	CH_3O-Ce (II)
1552-1555	v_{as}(OCO)	$HCOO^-/Ce^{4+}$
1576-1580	v_{as}(OCO)	$HCOO^-/Ce^{3+}$
2713-2718	δ(CH) + v_{sym}(OCO)	$HCOO^-/Ce^{4+}$- Ce^{3+}
2800-2816	$v_{sym}(CH_3)$	CH_3O-Ce (I) and (II)
2843	v_{sym}(CH)	$HCOO^-/Ce^{4+}$- Ce^{3+}
2865	$2\delta(CH_3)$	CH_3O-Ce (I)
2885-2888	$2\delta(CH_3)$	CH_3O-Ce (II)
2910-2913	$v_{as}(CH_3)$	CH_3O-Ce (I)
2922-2923	$v_{as}(CH_3)$ + $2\delta(CH_3)$	CH_3O-Ce (II)
2938-2941	δ(CH) + v_{sym}(OCO)	$HCOO^-/Ce^{4+}$- Ce^{3+}

Table S2: Bands frequencies assignment obtained during CO_2 adsorption over CeO_2-MOF and CeO_2-HSA[5–7]

Frequency (cm^{-1})	Assignment	Species
824-827,840	$\pi(CO_3)$	H-CO_3^- (*I* and *II*)
851-856	$\pi(CO_3)$	b-$CO_3^=$
1016-1029,1042-1050	$v_{sym}(CO_3)$	H-CO_3^- (*I* and *II*)
1080-1114	$v_{sym}(CO_3)$	poly-$CO_3^=$
1216-1217	δ(OH)	H-CO_3^-
1293	$v_{sym}(CO_3)$	b-$CO_3^=$
1342-1345	$v_{sym}(CO_3)$	poly-$CO_3^=$
1365-1369	$v_{sym}(CO_3)$	m-$CO_3^=$
1391-1410	$v_{sym}(CO_3)$	H-CO_3^- (*I* and *II*)
1465-1468	$v_{sym}(CO_3)$	poly-$CO_3^=$
1505-1516	$v_{as}(CO_3)$	m-$CO_3^=$
1520-1564	$v_{as}(CO_3)$	poly-$CO_3^=$
1579-1581	$v_{as}(CO_3)$	b-$CO_3^=$
1602-1610	$v_{as}(CO_3)$	H-CO_3^-
3618	v(OH)	H-CO_3^-

4. References

(1) Binet, C.; Daturi, M.; Lavalley, J.-C. IR Study of Polycrystalline Ceria Properties in Oxidised and Reduced States. *Catal Today* **1999**, *50* (2), 207–225. https://doi.org/10.1016/S0920-5861(98)00504-5.

(2) Binet, C.; Daturi, M. Methanol as an IR Probe to Study the Reduction Process in Ceria–Zirconia Mixed Compounds. *Catal Today* **2001**, *70* (1–3), 155–167. https://doi.org/10.1016/S0920-5861(01)00415-1.

(3) Jung, K. T.; Bell, A. T. An in Situ Infrared Study of Dimethyl Carbonate Synthesis from Carbon Dioxide and Methanol over Zirconia. *J Catal* **2001**, *204* (2), 339–347. https://doi.org/10.1006/jcat.2001.3411.

(4) Wu, Z.; Li, M.; Mullins, D. R.; Overbury, S. H. Probing the Surface Sites of CeO2 Nanocrystals with Well-Defined Surface Planes via Methanol Adsorption and Desorption. *ACS Catal* **2012**, *2* (11), 2224–2234. https://doi.org/10.1021/cs300467p.

(5) Busca, G.; Lorenzelli, V. Infrared Spectroscopic Identification of Species Arising from Reactive Adsorption of Carbon Oxides on Metal Oxide Surfaces. *Materials Chemistry* **1982**, *7* (1), 89–126. https://doi.org/10.1016/0390-6035(82)90059-1.

(6) Lavalley, J. C. Infrared Spectrometric Studies of the Surface Basicity of Metal Oxides and Zeolites Using Adsorbed Probe Molecules. *Catal Today* **1996**, *27* (3), 377–401. https://doi.org/10.1016/0920-5861(95)00161-1.

(7) Vayssilov, G. N.; Mihaylov, M.; Petkov, P. St.; Hadjiivanov, K. I.; Neyman, K. M. Reassignment of the Vibrational Spectra of Carbonates, Formates, and Related Surface Species on Ceria: A Combined Density Functional and Infrared Spectroscopy Investigation. *The Journal of Physical Chemistry C* **2011**, *115* (47), 23435–23454. https://doi.org/10.1021/jp208050a.
